# The NO-cGMP-PKG signal transduction pathway is involved in the analgesic effect of early hyperbaric oxygen treatment of neuropathic pain

**DOI:** 10.1186/s10194-017-0760-z

**Published:** 2017-05-03

**Authors:** Yuanyuan Ding, Peng Yao, Tao Hong, Zhenkai Han, Baisong Zhao, Weimin Chen

**Affiliations:** 1 Department of Pain Management, Shengjing Hospital of China Medical University, Shenyang, 110004 China; 2 Department of Anesthesiology, Shengjing Hospital of China Medical University, Shenyang, 110004 China; 3 Department of Anesthesiology, Guangzhou Women and Children’s Medical Center, Guangzhou, China

**Keywords:** Hyperbaric oxygen, Neuropathic pain, Nitric oxide, Cyclic GMP, Protein kinase G-1, Chronic constriction injury

## Abstract

**Background:**

Hyperbaric oxygen (HBO) has the potential to relieve neuropathic pain. The purpose of this study was to determine whether the NO-cGMP-PKG signaling pathway is involved in the analgesic effects of early hyperbaric oxygen treatment of neuropathic pain in rats.

**Methods:**

Rats were randomly grouped for establishment of chronic constriction injury (CCI) models. Intrathecal catheters were inserted and 2.5ATA HBO therapy was administered from day 1 post-surgery for 60 minutes daily, continuously for 5 days; menstruum NS, DMSO, NO synthase(NOS) nonspecific inhibitor (L-NAME), soluble guanylyl cyclase(sGC) inhibitor (ODQ) and protein kinase G(PKG) inhibitor (KT5823) were administered intrathecally 30 minutes prior to HBO therapy. Pain-related behaviors in rats were observed at specific time points. Western blot and real-time RT-PCR were used to observe the expressions of PKG1 mRNA and protein in the spinal dorsal horn.

**Results:**

Compared with the CCI group, HBO could significantly relieve mechanical and thermal hyperalgesia in rats. After intrathecal administration of L-NAME, ODQ and KT5823, effects of HBO on relieving hyperalgesia in rats were reversed (*P* < 0.05 vs. HBO), and expression of PKG1 mRNA and protein decreased in the spinal dorsal horn of the animals (*P* < 0.05 vs. HBO).

**Conclusions:**

Early HBO therapy could significantly improve symptoms of hyperalgesia of neuropathic pain in rats, possibly via activation of the NO-cGMP-PKG signaling transduction pathway.

## Background

International Association for the Study of Pain (IASP) [1] defined the neuropathic pain (NP) as pain induced by direct injury or dysfunction of the central or peripheral nervous systems. NP is a highly prevalent and etiologically complex disease. All factors including metabolic (e.g. diabetic neuropathy), infectious (e.g. postherpetic neuralgia), autoimmune (e.g. multiple sclerosis), vascular (e.g. stroke), nervous (e.g. trigeminal neuralgia) and cancerous can lead to NP. The disease is chronic and progressive, and is often characterized by spontaneous pain, hyperalgesia and allodynia [2–4]. NP currently lacks an effective management strategy, and has become one of the most challenging problems in clinical medicine.

Hyperbaric oxygen (HBO) has a strong neuroprotective effect, and is widely used in the treatment of a variety of neurological diseases. Clinically, some advances in the treatment of chronic pain have been reported [5–7]. In animal models, HBO has been reported to effectively relieve inflammatory pain [8]. Studies have reported that HBO could effectively relieve pain of two neuropathic pain models [9]. Previous studies noted that single HBO administration [10] could relieve mechanical allodynia and thermal hyperalgesia in CCI rats, but repeated hyperbaric oxygen therapy and follow-up mechanism was not clear. Furthermore, we found that HBO could significantly decrease the expression of iNOS and nNOS in the spinal dorsal horn on the operative side, with no obvious change of eNOS expression. However, several studies revealed that HBO could improve circulation by increasing NO production, resulting in pain alleviation. Although results were contradictory, NO played an important role in neuropathic pain remission after HBO therapy. A large number of studies have demonstrated that the NO-cGMP-PKG signaling pathway was involved in hyperalgesia of inflammatory and neuropathic pain [11, 12], and was suggested to play a vital role in the transmission of nociceptive signals.

The NO-cGMP-PKG signaling pathway is involved in both pain induction [13] and analgesia [14, 15]. Lindsay P et al. found that HBO could significantly relieve abdominal pain in the acute inflammatory abdominal pain mouse model induced by intraperitoneal injection of 1% acetic acid. In addition, its acute anti-nociceptive effect was due to activation of NO-cGMP-PKG-KATP channels [16]. However, the role of the NO-cGMP-PKG signaling pathway in early HBO treatment of neuropathic pain has not previously been elucidated.

Therefore, in this study, the CCI model was selected for early HBO treatment. After different pressures of hyperbaric oxygen therapy, we preferred 2.5ATA for 5 consecutive days of treatment for the experimental treatment conditions [17]. Whether HBO is involved in regulation of hyperalgesia of neuropathic pain via the NO-cGMP-PKG signaling pathway was evaluated by intrathecal administration of NOS nonspecific inhibitor (L-NAME), sGC inhibitor (ODQ) and PKG inhibitor (KT582) at onset of neuropathic pain.

## Methods

### Animal

Healthy male Sprague-Dawley (SD) rats weighing 270–290 g were selected and kept at a room temperature of 23–25 °C and a relative humidity of 40–60% in a natural light environment. Animals were housed alone, with food and water freely available. Rats were numbered for surgeries of CCI and intrathecal catheter insertion, and randomly divided into 7 groups: CCI group; HBO group; HBO + NS group (NS10μL); HBO + DMSO group (DMSO10μL); HBO + L-NAME group (L-NAME100μg/10 μL); HBO + ODQ group (ODQ10μg/10 μL); HBO + KT5823 group (KT5823 500 ng/10 μL). 2.5ATA hyperbaric oxygen therapy was performed from day 1 after surgery, for 60 minutes every day, continuously for 5 days; Menstruums and inhibitors were intrathecally administered 30 minutes prior to hyperbaric oxygen therapy. All animals were purchased from the Animal Center of Shengjing Hospital affiliated to China Medical University. All animal experiments in this study were under approved protocols of the Institutional Animal Ethics Committee of the China Medical University (2016PS063K) and carried out in accordance with the Guidelines for the Care and Use of Laboratory Animals of the National Institutes of Health.

### Establishment of the neuropathic pain model (CCI)

The CCI model of sciatic nerve injury was established according to methods reported by Bennett and Xie [18]. Rats were anesthetized with an intraperitoneal injection of 10% chloral hydrate (300 mg/kg). A lateral incision was performed at the left hindlimb, the sciatic nerve trunk was located and relaxation ligation was performed using 4 silks (4-0#) with an interval distance of 1 mm. Adequate muscle contraction in the control area of the sciatic nerve with compressed adventitia was observed. Layered skin suturing was then performed.

### Subarachnoid catheter insertion

Rats were anesthetized with an intraperitoneal injection of 10% chloral hydrate. With the rats in the prone position, fur was shaved and skin was disinfected. The L6 level of the spinal cord was identified as the middle point of connection between both sides of the iliac spine. The L3-4 was found and a longitudinal incision with a length of 2 cm was performed. The dorsal fascia of the spine was incised and the interspinous ligaments at levels L3-4 and L4-5 were removed. The interspinous space between L3 with L4 was exposed and the fascia and muscle tissue were subsequently carefully separated. The spine of the rats was curled forward as far as possible, and the ligamentum flavum was gently perforated. The lamina of L3 was lifted upward using a towel clamp, and a PE-10 catheter was inserted into the rostral side at a depth of 2 cm. Proper catheter insertion into the subarachnoid space was confirmed if clear cerebrospinal fluid flowed slowly out of the catheter. The head of the catheter was exposed from the nape at a length of 1 cm. Rats were observed for 24 hours. Animals without noticeable sensory and motor disorders were selected as subjects.

### Hyperbaric oxygen therapy

Hyperbaric oxygen treatment was administered to all rats in the HBO group on the first day after surgery. Every time prior to entering the cabin (DS400-IV, Weifang Huaxin Oxygen Industry Co.,Ltd., Shandong, China), fresh lime was arranged at the bottom of the hyperbaric oxygen chamber for reduction of water vapor and CO_2_. Pure oxygen was used for washing the cabin to maintain an oxygen concentration of >90%. After the animal was placed into the cabin, pressure was slowly increased to 2.5ATA at a velocity of 0.125ATA/min; cabin conditions were maintained for 60 minutes. After treatment, pressure was reduced to normal within 20 minutes. Behavior of animals in the cabin during treatment was observed carefully. Rats in the CCI group were also placed into the cabin to simulate other treatment processes and environmental conditions while not being administered hyperbaric oxygen.

### Observation of pain-related behavior

After surgery, all animals were housed alone in a cage. Gait, post-operative position of the left limb, with or without autophagy and pain-related behaviors of rats were recorded.

### Spontaneous foot contractions:

Rats were placed in a transparent glass box and habituated for 15 min. Rats were then allowed to freely roam and the number of spontaneous contractions of the left hind limbs within five minutes was observed.

### Mechanical withdrawal threshold (MWT) measurement:

The MWT was measured using an “up and down” method, with Von Frey filaments (Stoelting, Chicago, IL, USA) for determination. Measurement was performed at 10:00–12:00 a.m.daily. Rats were placed on an elevated test stand covered with a transparent organic glass. After a 15 minute habituation, the time of disappearance of grooming and exploratory activity was noted. Rat feet were graduated stimulated by using a scaled Von Frey filament, with 0.14–15 g filaments gradually pressurized until they bended. Stimulation lasted for 3 seconds during each measurement, with an interval of 15 seconds between measurements. The lowest value of Von Frey filament stimulation, potentially resulting in raising of the rat paw for over 5 times, was defined as the mechanical withdrawal threshold. A painless response was considered to result in a lack of foot contraction after stimulation with a 0.15 g Von Frey filament. Foot contraction responses caused by body movement were not recorded as positive responses.

### Thermal withdrawal latency(TWL) determination:

Thermal pain stimulation (Youer Equipment Scientific Co., Ltd., Shanghai, China) was used for TWL determination. Rats were placed in a testing frame. After habituation for 15 minutes, an optical source was targeted on the left hind foot, and a thermal pain stimulator (light source) and timer were activated. When rat feet were raised, the timer was stopped and time recorded. TWL was calculated as the latency from the beginning of thermal pain stimulator irradiation to the shrinkage or significant movement of the foot. The average value for each foot was calculated from averaging results of three sequential tests, performed at intervals of 10 minutes. In order to prevent scalding of rat feet, an upper latency limit of 30 seconds was established. If rat feet did not contract after irradiation for over 30 seconds, thermal stimulation was terminated automatically, and the duration recorded was 30 seconds.

### Sampling

After the pain threshold test, rats were anesthetized with 10% chloral hydrate solution (300 mg/kg) and then decapitated on the 7th day after surgery. Six rats were selected from each group. Under aseptic conditions, L4-6 spinal cord levels were carefully removed and the dorsal horns on the operative side were quickly sliced and stored in liquid nitrogen at −80 °C.

### Western blot

Cell lysate was added into the −80 °C frozen spinal cord tissue and rapidly homogenized on ice and centrifuged at 4 °C. The supernatant was aliquot and the concentration of protein was determined via the BAC method. Samples containing 80 μg of protein were loaded into 10% sodium dodecyl sulfate polyacrylamide gel electrophoresis (SDS-PAGE), proteins were then electrotransferred to a polyvinylidene fluoride (PVDF) membrane (Amersham Bio-sciences, Freiburg, Germany) and blocked at room temperature in a blocking solution consisting of TBST (Tris-buffer saline containing 0.05% Tween-20). Membranes were then incubated overnight at 4 °C with primary antibodies of anti-PKG1 (Cell Signaling Technology, Boston, MA, American, 1:1000) and anti-GAPDH (Sigma Chemical Co, St. Louis, MO, USA, 1:1000) dissolved in TBST containing 5% nonfat dry milk. Membranes were then washed with TBST and incubated with HRP labeled secondary antibody (ComWin Biotech, Beijing, China, 1:5000) diluted with 5% nonfat dry milk under room temperature for one hour. The membrane was detected by reaction with the Amersham ECL-Plus reagent (Amersham Bio-sciences, Freiburg, Germany).

### Real-time PCR

Total RNA was extracted according to instructions provided with the utilized Trizol reagent (Invitrogen, Carlsbad, CA, USA); RNA concentration and content was evaluated via spectrophotometer, and cDNA was synthesized by reverse transcription of the RNA. The primer sequences of PKG1 were as follows: upstream primer 5'-AGT GGT TTG AGG GCT TTA-3'; downstream primer 5'-ATG TCC CAG CCT GAG TTG-3'. The primer sequences of GAPDH were as follows: upstream primer 5'-ATG ACT CTA CCC ACG GCA G-3'; downstream primer 5'-GGA AGA TGG TGA TGG GTT TC-3'. PKG1 and GAPDH were amplified using a real-time PCR thermal cycler (Takara, Kyoto, JPN). Experimental conditions were as follows: 95 °C for 30 seconds,95 °C for 5 seconds,60 °C for 30 seconds, and 72 °C for 10 seconds. A total of 45 cycles were performed. The dissolution curve of the product was finally analyzed.

### Statistical analysis

SPSS16.0 software was used for data analysis. Measured data were represented as mean ± standard deviation. One-way analysis of variance (ANOVA) was applied to compare differences between groups; the least significant difference (LSD) was used for post Hoc analysis. *P* < 0.05 was considered statistically significant.

## Results

### Observation of pain-related behavior

After successful establishment of a CCI model, left limb claudication was noted in rats on the third day after surgery. In addition, other behaviors suggesting pain (such as foot curling, frequent licking, etc.) were observed. Claw contracture and joint stiffness were subsequently noted. After early 2.5ATA hyperbaric oxygen treatment, almost no limb claudication was observed in rats as they moved about. Left limbs were capable of supporting their body weight, and the toes stayed separated. Intrathecal injection of L-NAME, ODQ and KT5823 could reverse analgesic effects of HBO, leading to obvious neuropathic pain symptoms again.

**Fig. 1:**
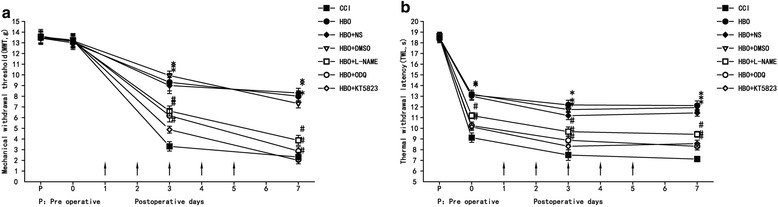
MWT and TWL in all rats bothpreoperatively and postoperatively. HBO treatment was performed once daily for five consecutive days beginning from the first day after CCI, as indicated by arrows. NS, DMSO, L-NAME, ODQ and KT5823 were intrathecally delivered once daily half an hour after HBO treatment. **a** Changes on mechanical allodynia. **b** Changes on thermal hyperalgesia. Comparison of TWL and MWT in rats: *P < 0.05 vs. CCI; # *P* < 0.05 vs. HBO. *N* = 6 rats for each group. HBO treatment at early stages alleviates neuropathic pain; L-NAME, ODQ and KT5823 can reverse HBO effect

Compared with the CCI group, MWT and TWL were significantly improved in the HBO, NS, and DMSO groups (*P* < 0.05). There was no significant difference between these groups (*P* > 0.05). Compared with the HBO group, MWT and TWL were significantly decreased after intrathecal injection of L-NAME, ODQ and KT5823 (*P* < 0.05). Intrathecal injection of these substances altered the state of hyperalgesia in CCI rats after HBO treatment, suggesting that they played a role in antagonizing the analgesic effect of HBO in neuropathic pain (Fig. 1).

### Western blot

**Fig. 2:**
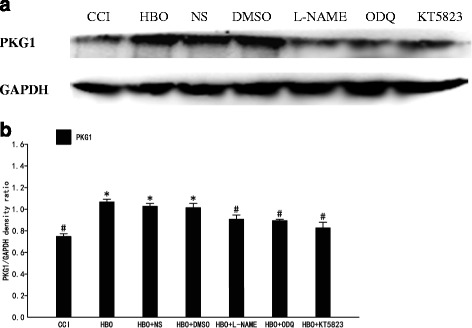
Western blot of PKG1 expression in the dorsal horn spinal cord. **a** The upper panel displays the bands of PKG1 and GAPDH. **b** The lower panel displays statistical results of Western blot analysis. Data are shown as mean ± S.E.M, *n* = 6. *P < 0.05 vs. CCI; # *P* < 0.05 vs. HBO. PKG1 protein expression in the dorsal horn spinal cord was significantly increased after HBO treatment, while when L-NAME, ODQ and KT5823 were intrathecally delivered, PKG1 protein expression was decreased

Western Blot was used to detect expression of PKG1 protein in the dorsal horn of the rat spinal cord (Fig. 2). Compared with the CCI group, expression of PKG1 protein in the HBO group was significantly increased (*p* < 0.05). Compared with the HBO group, expression of PKG1 protein in the NS and DMSO groups showed no significant changes. Compared with the HBO group, expression of PKG1 in the L-NAME, ODQ and KT-5823 groups was significantly decreased (*p* < 0.05). These results suggest that increased expression of PKG1 was induced by HBO. PKG1 protein expression in the spinal cord induced by HBO was inhibited by intrathecal injection of nonselective NOS, sGC and PKG1 inhibitors, suggesting that HBO may exert its analgesic effects via activation of the NO-cGMP-PKG signaling pathway.

### Real-time fluorescence quantitative PCR

**Fig. 3:**
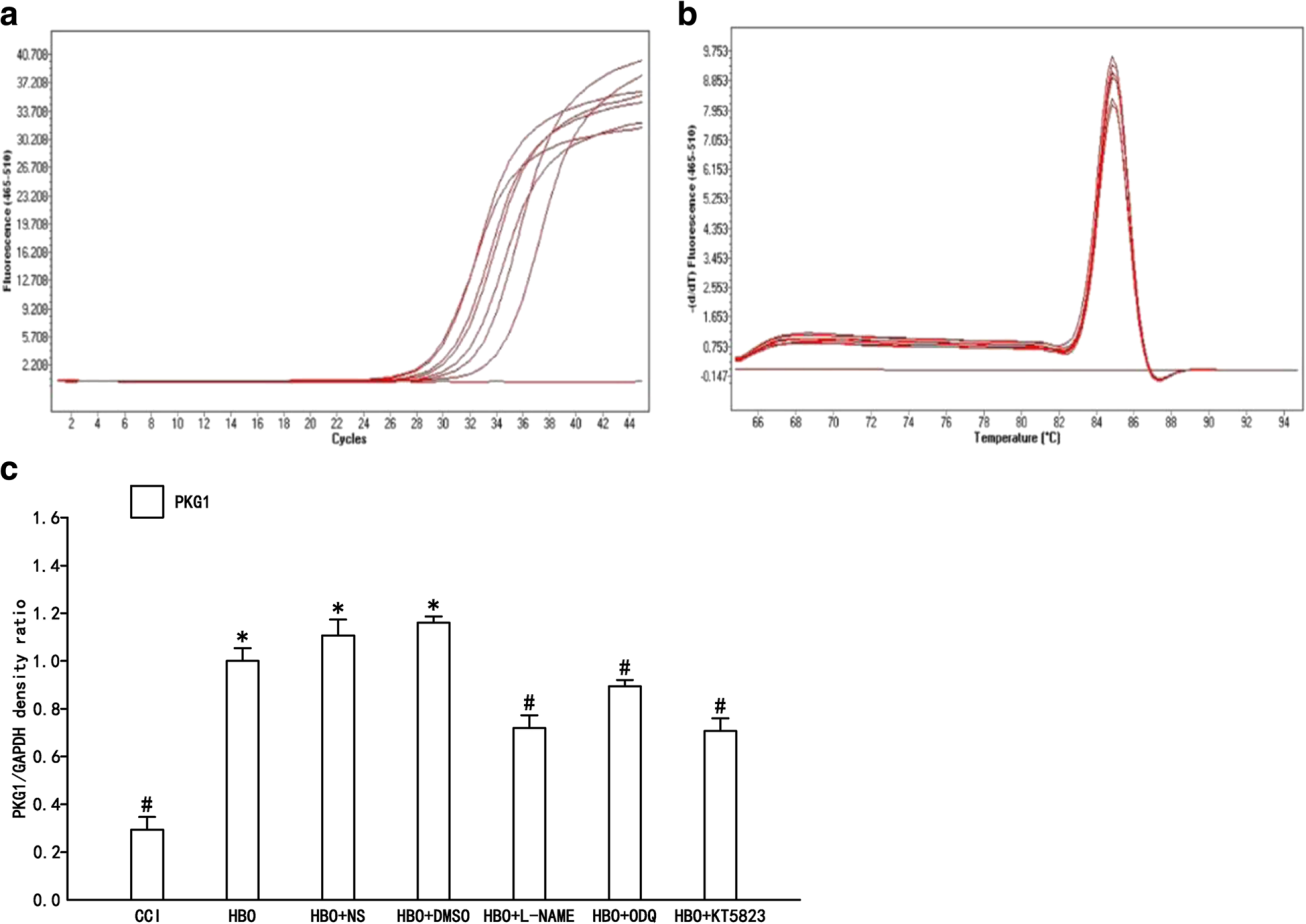
RT-PCR analysis of PKG1 mRNA content in the dorsal spinal horn. Statistical results: **a** Amplification curves; **b** Dissociation curves; **c** Relative PKG1 mRNA expression (PKG1/GAPDH). Data are shown as mean ± S.E.M, *n* = 6. *P < 0.05 vs. CCI; # *P* < 0.05 vs. HBO. HBO increased the expression of PKG1 in the dorsal horn of the CCI model. After intrathecal injection of L-NAME, ODQ and KT5823, PKG1 expression significantly decreased compared with groups treated with HBO

Real-time (RT) PCR was used to detect expression of PKG1 mRNA in the dorsal horn of the spinal cord in each group (Fig. 3). Compared with the CCI group, expression of PKG1 mRNA in the HBO group was significantly increased (*p* < 0.05). Compared with the HBO group, expression of PKG1 mRNA in NS and DMSO groups revealed no significant change. Compared with the HBO group, expression of PKG1 mRNA in the L-NAME, ODQ and KT-5823 groups were significantly decreased (*P* < 0.05). These results suggest that increased expression of PKG1 mRNA was induced by HBO treatment. PKG1 gene expression induced by HBO treatment in the spinal cord was inhibited by intrathecal injection of nonselective NOS, sGC and PKG1 inhibitors; these data further support NO-cGMP-PKG signaling pathway involvement after HBO treatment of neuropathic pain.

## Discussion

The causes of neuropathic pain are numerous, and the corresponding mechanisms are complicated. Conventional therapeutic drugs have no obvious effect [19], while neural interventional therapies, such as transcutaneous electrical nerve stimulation (TENS) and spinal cord stimulation (SCS) are often too expensive for patients to afford. Therefore, it would be highly beneficial to find a simple, economical, convenient and effective treatment modality.

The CCI model possesses characteristics of peripheral and central sensitization, similar to clinical features of NP, and has been widely used in studies on pain [20]. Our previous study found that early HBO treatment could effectively alleviate mechanical and thermal hyperalgesia. This effect of neuropathic pain alleviation could persist until cessation of HBO therapy, suggesting that protective pathways were activated, although mechanism(s) remained unclear.

The analgesic effect of early HBO treatment was reported to potentially reduce the production of tumor necrosis factor-α (TNF-α) [21], and tumor necrosis factor gene polymorphism was also involved [22]. The long-term analgesic effect was suggested to be related to induction of release of NO dependent opioid peptides [23], and NO could inhibit intercellular adhesion molecule-1 (ICAM-1), soluble ICAM-1 and interleukin-4 [24]. While the NO-cGMP-PKG signaling transduction pathway was reported to be involved in the development of damage-induced hyperalgesia [25, 26], studies on its role in NP are scarce. We previously found that HBO could regulate the expression of iNOS and nNOS levels in the spinal cord [10]. HBO can significantly increase the production of NO, particularly in the striatum, brain stem, cerebellum and spinal cord [27, 28]. NO can activate soluble guanylyl cyclase (sGC) to cyclize GTP producing cyclic guanoine monophosphate (cGMP), and cGMP can activate cGMP-dependent PKG. PKG can then exert biological effects via phosphorylation of target protein. PKG plays a central regulating role in the NO-cGMP-PKG signaling pathway. Studies found that PKG can further activate ATP sensitive potassium channels and promote the efflux of potassium ions, further enhancing the analgesic effect [15].

In this study, inhibitors of the NO-cGMP-PKG signal transduction pathway were administered at an early stage of HBO treatment, and analgesic effects on neuropathic pain were observed. The analgesic effect of HBO was blocked by the NOS non-selective inhibitor L-NAME. Therefore, we speculated from inhibition of HBO-induced analgesic effects by the sGC inhibitor ODQ that the NO-cGMP pathway was involved in influencing neuropathic pain symptomology. KT-5823, a specific inhibitor of PKG, inhibited the analgesic effect of HBO on NP, consistent with reports regarding the treatment of early acute inflammatory pain [16]. Our results suggest that the analgesic effect of early HBO treatment was mediated by NO and magnified by GC activation; the activation of the NO-cGMP-PKG pathway played a prominent role in analgesia brought on by early HBO treatment; Hyperbaric oxygen may provide a new target for the treatment of neuropathic pain.

In previous experiments, we observed that HBO could reduce the generation of iNOS and nNOS. Pain should therefore be relieved by reducing generation of NO. However, administration of NO-cGMP-PKG pathway inhibitors proved surprising. NO content increased after HBO treatment, possibly due to HBO treatment providing a substrate (oxygen) for NO synthesis, in turn promoting synthesis of NO. HBO treatment could reduce NO loss, indirectly increasing NO content. HBO treatment increased blood oxygen content and the partial pressure of oxygen, reversing the usual inhibition of NOS activity under hypoxic conditions. In preliminary experiments, we found that when HBO treatment was abandoned at a late stage in the CCI group, the pain threshold decreased, resulting in hyperalgesia. We therefore hypothesized that HBO treatment produced a biphasic effect on NP. Its analgesic effect was promoted via activation of the NO-cGMP-PKG pathway at the early stage of treatment, while the reduction of iNOS and nNOS production inhibited the activation of glial cells, reducing release of inflammatory factors, further interrupting pain transmission. Although the NO-cGMP-PKG signaling transduction pathway was involved in the analgesic effect of HBO treatment of NP, it was not the sole mechanism of the exerted analgesic effect. Zelinski et al. found that HBO treatment could increase endogenous opioid levels [23]. Zhao B et al. found that HBO could reduce neuropathic pain and inflammatory responses by modulating the Kindlin-1/Wnt-10a signaling pathway [29], reflecting the complexity of mechanisms involved in HBO treatment relieving neuropathic pain.

PKG is an important effector of cGMP, with a variety of physiological functions [30]. It has two subtypes, PKG1 and PKG2; tissue distribution of different PKG subtypes varies [31]. PKG1 is widely distributed throughout the nervous system [32], while PKG2 exists in the serosa and in the kidney, cerebellum, and mucosal layer [33]. Therefore, PKG1 was selected to detect changes of PKG1 after early HBO treatment. Experimental results of RT PCR and Western blot confirmed that HBO could increase expression of PKG1 in the spinal cord, suggesting that PKG1 played an important role in the analgesic effect exerted by HBO treatment. However, much controversy about the mechanism of action of PKG1 exists, and the role of PKG in NP remains unclear. However, many studies have reported PKG1 to exert an analgesic effect in the setting of early HBO treatment [34]. Administration of NOS nonspecific inhibitor (L-NAME), sGC inhibitor (ODQ) and PKG inhibitor (KT582) reversed effects of HBO treatment in the CCI model of hyperalgesia, suggesting that the anti-nociceptive effect of HBO treatment may be mediated by the NO-cGMP-PKG signal transduction pathway. Subsequent sites of interaction are topics for future research.

## Conclusion

In conclusion, early HBO relieves mechanical allodynia and thermal hyperalgesia in CCI rats via activation of the NO-cGMP-PKG signaling pathway. Early HBO therapy increased the expression of PKG1 in the spinal dorsal horn, presumably key in pain mediation.

## Abbreviations

CCI: Chronic constriction injury
cGMP: cyclic guanosine monophosphate
eNOS: endothelial nitric oxide synthase
HBO: Hyperbaric oxygen
iNOS: inducible nitric oxide synthase
L-NAME: N-nitro-L-arginine methyl ester
MWT: Mechanical withdrawal threshold
nNOS: neuronal nitric oxide synthase
NO: Nitric oxide
NP: Neuropathic Pain
PKG: protein kinase G
SCS: Spinal Cord Stimulation
SD: Sprague–Dawley
sGC: soluble gunylte cyclse
TENS: Transcutaneous Electrical Nerve Stimulation
TWL: Thermal withdrawal latency
